# Systematic Review of Breast Cancer Biology in Developing Countries (Part 2): Asian Subcontinent and South East Asia

**DOI:** 10.3390/cancers3022382

**Published:** 2011-05-13

**Authors:** Riyaz Bhikoo, Sanket Srinivasa, Tzu-Chieh Yu, David Moss, Andrew G Hill

**Affiliations:** 1 Department of Surgery, South Auckland Clinical School, University of Auckland, Auckland 1640, New Zealand; E-Mails: sanket.srinivasa@middlemore.co.nz (S.S.); wendells9@gmail.com (T.-C.Y.); andrew.hill@middlemore.co.nz (A.G.H.); 2 Department of Surgery, Middlemore Hospital, Auckland 1640, New Zealand; E-Mail: david.moss@middlemore.co.nz (D.M.)

**Keywords:** breast cancer, breast neoplasm, ethnicity, developing countries, low income countries

## Abstract

There has been no systematic appraisal of ethnicity-based variations in breast cancer (BC) biology amongst women from developing countries. A qualitative systematic review was conducted of breast cancer size, stage, grade, histological type, extra-mammary involvement, hormone receptor status as well as patient demographics. This review includes patients from Africa, the Middle East, Eastern Europe, Mexico, the Caribbean and South America. BC in these regions present at an earlier age with large aggressive tumours. Distant metastases are frequently present at the time of diagnosis. African women have a higher frequency of triple negative tumours. Over half of Middle Eastern women have lymph node involvement at the time of diagnosis. Despite experiencing a lower incidence compared to the Ashkenazi Jewish population, Palestinian women have poorer five-year survival outcomes. The majority of women from Mexico and South America have stage two or three disease whilst over sixty percent of women from Eastern Europe have either stage one or stage two disease. The biological characteristics of BC in the Caribbean cannot be fully assessed due to a paucity of data from the region. BC amongst the developing world is characterised by an early peak age of onset with aggressive biological characteristics. Strategies that improve breast cancer awareness, address amenable risk factors and improve early detection are essential.

## 1. Introduction

There is a paucity of data regarding Breast cancer (BC) in the developing world. We have previously reported on the BC characteristics from women residing in Africa, the Middle East, Eastern Europe, Mexico, the Caribbean and South America [1]. Here we present the results for the Asian Subcontinent and South East Asia.

## 2. Methods

The methods and search strategy employed was the same as described in the first paper of this series [1].

## 3. Results

A flow diagram (QUROUM statement) outlining the process by which papers were selected for further evaluation is presented in the first manuscript of this series [1].

An overview of the biological features of BC within Pakistan, India and Sri Lanka is presented in Table 1.

### 3.1. Asian Subcontinent

#### 3.1.1. Pakistan

##### 3.1.1. Background

Pakistan has one of the highest reported incidence rates of BC in Asia with an ASR of 54 per 100,000 reported in Karachi South [2]. Bhurgri *et al.* found that 60% of newly diagnosed cases of BC occurred in those less than 50 years of age [2]. Data on tumour biology can be found in Table 1.

##### 3.1.2. Hormone receptor status

Her-2 positivity was found in 25% and 31% of patients from Karachi and Rawalpindi respectively [3,6]. Azizun-Nisa *et al.* found ER and PR expression to be significantly lower in Her-2 positive tumours compared with Her-2 negative tumours (ER 84% *vs.* 70%; PR 92% *vs.* 78%) [3]. Analysis of 315 breast tumours with IDC found over expression of p53 in 55% of cases with p53 positivity associated with regional lymph node involvement and distant metastases [11].

#### 3.1.2. India

##### 3.1.2.1. Background

For the period 2001–2004, BC was the commonest cancer amongst females in many of India's urban cities such as Mumbai and Delhi [12]. However, in certain rural communities the measured incidence is less than that of cervical cancer [12].

##### 3.1.2.2. Tumour biology

In New Delhi, Saxena *et al.* found that 62% (230/369) of women had clinical stage three disease, 28% (105/369) had stage two disease and eight percent (29/369) had stage four disease [7]. The mean age of cases with stage IIA and IV disease was 45 and 51 years respectively [7].

##### 3.1.2.3. Hormone receptor status

Desai *et al.* reported on 798 patients where the median age was 48 years and found 33% were ER+ and 46% were PR+ [13]. Data on combined receptor profiles found 25% were ER+/PR+, 7% were ER+/PR−, 21% were ER−/PR+ and 47% were ER−PR− [13]. ER and PR immunoreactivity increased with advancing age and correlated with the presence of elastosis [13]. Higher grades of IDC were associated with reduced ER/PR positivity while the presence of necrosis and lymphovascular invasion was inversely related to hormone receptor status [13]. Twenty percent of patients were found to be Her-2 positive [13].

#### 3.1.3. Sri Lanka

##### 3.1.3.1. Background

BC is the most prevalent cancer amongst Sri Lankan women [14]. Lokuhetty *et al.* reported a mean age at presentation of 52 years with 32% of patients between 50 to 59 years of age [9].

##### 3.1.3.2. Hormone receptor status

Lokuhetty *et al.* found that 32% (254/802) of cases were ER + while 15% (96/662) were Her-2 positive. [9] Well differentiated tumours were more likely to be ER+ and Her-2 negative [9]. In a study of 151 patients, Mudduwa *et al.* found that 54% (82/151) and 52% (75/145) of tumours were negative for ER and PR respectively [10]. Nineteen percent (26/136) of patients were positive for Her-2 and of these 69% were ER negative and 62% were PR negative [10]. Ratnatunga *et al.* found ER, PR and Her-2 (3+) positivity was reported in 53% (66/124), 50% (62/124) and 15% (18/123) of cases respectively [15]. Furthermore, 44% (55/124) were ER+/PR+, 41% (51/124) were ER−PR−, 9% (11/124) were ER+/PR− and 6% (7/124) were ER−/PR+ [15].

#### 3.1.4. Nepal

##### 3.1.4.1. Background

Pradhan *et al.* reported on the fine needle aspirate (FNA) findings of 2246 female patients presenting with breast lumps with a mean age of 47 years [16].

##### 3.1.4.2. Tumour biology

The two most common findings were fibroadenosis [43% (975/2246)] followed by BC [15% (348/2246)] [16]. Of those with malignancy, 96% of patients were greater than 30 years of age [16]. Ductal carcinoma was reported in 97% (338/348) of cancer FNA specimens with mucinous carcinoma and medullary carcinoma accounting for one percent (3/348) each [16]. Furthermore, 52 cases suspicious of malignancy were evaluated later by histology and 63% (27/43) were found to be malignant [16].

Sharma *et al.* reported histology data for 23 cases of which 91% (21/23) were IDC with single cases of medullary and lobular carcinoma reported [17]. Stage three cancer was reported in 55% (12/22) of cases followed by stage two at 27% (6/22) and stage one and four disease at nine percent each (2/22) [17].

##### 3.1.4.3. Hormone receptor status

Sharma *et al.* found that 44% (4/9) of patients were either ER+ or PR+ [17].

### 3.2. South East Asia

The tumour characteristic of BC amongst women from South East Asia has been outlined in Table 2.

### 

#### 3.4.1. China

##### 3.4.1.1. Background

Data from Shanghai for the year 2005 showed that BC was the commonest cancer in females [26].

##### 3.4.1.2. Tumour biology

###### Tianjin

Liu *et al.* showed the detection rate of early BC (stages zero and one) increased from 12% (1981–1985) to 16% (1996–2000) while the frequency of stage three cancer decreased from 25% to 17% over the same period [18]. Over the 20-year period, the rates of axillary lymph node involvement decreased from 52% to 46% while the rates of local recurrence/distant metastatic disease declined from 28% to 14% [18].

###### Changchun

Gao *et al.* found 53% (76/144) of patients were pre-menopausal [19]. The tumour size ranged from 3 mm to 146 mm, with a median diameter of 25 mm [19].

###### Shanghai

Zhang *et al.* reported on 476 cases of IDC and found that 15% (72/476) of cases were grade one, 45% (216/476) were grade two and 39% (188/476) were grade three [27].

##### 3.4.1.3. Hormone receptor status

Gao *et al.* found that 57% (82/144) of tumours were ER+ while 51% (74/144) were PR+ [19]. Expression of c-erbB-2 (Her-2) and p53 were observed in 40% (57/144) and 34% (49/144) of cases respectively [19]. Twenty two percent of patients had positive p53 antibodies which were associated with higher clinical stage, lymph node metastases, negative ER expression and positive c-erbB-2 (Her-2) status [19]. Yuan *et al.* found 24% (305/1280) of patients had triple-negative (ER/PR/Her-2) BC and this was commonly seen in younger patients [28].

#### 3.4.2. Taiwan

##### 3.4.2.1. Background

In 2006, BC was the commonest cancer amongst Taiwanese females and the fourth leading cause of cancer related death [29].

##### 3.4.2.2. Tumour biology

In a study of 1028 patients younger than 50 years of age, Lin *et al.* found 92% of cases were IDC with 22% reported as grade one, 55% as grade two and 23% as grade three (Table 2) [20]. Patients older than 50 years had fewer grade one cancers (18% *vs.* 22%) and a greater frequency of grade three tumours (30% *vs.* 23%) [20]. No significant difference in tumour histological type, cancer stage or nodal involvement was observed between the two age groups [20].

##### 3.4.2.3. Hormone receptor status

Lin *et al.* found patients younger than 50 years had significantly more ER+ (75% *vs.* 63%) and PR+ (47% *vs.* 33%) tumours than those older than 50 years [20]. Both age groups had similar expression for Her-2 (2+) (20% *vs.* 21%) [20]. Patients less than 50 years had more ER+/PR+ (51% *vs.* 31%), Luminal A (67% *vs.* 57%) and Luminal B tumours (10% *vs.* 8%) and a lower prevalence of ER−/PR− (23% *vs.* 36%), Her-2+/ER− (10% *vs.* 14%) and basal like tumours (9% *vs.* 17%) [20].

#### 3.4.3. South Korea

##### 3.4.3.1. Background

For the period 2003 to 2005, BC was the leading cancer amongst Korean females (15%) [30]. Ahn *et al.* reported on the chronological changes of Korean BC between 1996 and 2004 and found the number of registered cases had risen from 3801 to 9667 (154% rise) [21]. The median age at presentation remained constant at 47 years [21].

##### 3.4.3.2. Tumour biology

Between 1996 and 2004, Ahn *et al.* found the frequency of stage zero cancer increased from four percent to 10% while stage one cancer increased from 20% to 36% [21] The frequency of cancer stages two to four declined from 76% to 55% [21]. The incidence of ductal carcinoma in situ increased from four percent to 10% over the same period [21]. Choi *et al.* compared the biological features of early onset BC (before 45 years) between women from Korea and the United States of America (USA) and found the former had a larger mean tumour size (28 mm *vs.* 21 mm) with no significant differences in histological type and number of lymph nodes involved [31].

##### 3.4.3.3. Hormone receptor status

Son *et al.* reported ER and PR positivity in 57% and 51% of cases respectively [32]. Choi *et al.* reported that Korean women had greater expression of Her-2 receptors at 48% (28/59) compared to Caucasian women from the USA at 16% (9/57) [31] Expression of ER, PR, p53 and cyclin D1 was not statistically significant between the two groups [31].

Rhee *et al.* reported that 20% (136/683) of patients had triple negative breast tumours (negative for ER/PR/Her-2) and this was correlated with a younger age (<35 years), shorter relapse free survival and more aggressive clinicopathological characteristics as evidenced by higher rates of p53 and Ki67 expression, negative bcl-2 expression and greater positivity for epidermal growth factor receptor [33].

#### 3.4.4. Thailand

##### 3.4.4.1. Background

BC is the second most common cancer diagnosed in Thai women [34]. For the period 1998–2000, the estimated incidence was 21 per 100 000 while the peak age-specific incidence was at 45 years [34]. Lertsanguansinchai *et al.* reviewed 399 patients with BC and reported a mean age of 50 years [35].

##### 3.4.4.2. Tumour biology

IDC was reported between 76% and 91% of cases across nine Thai registries, followed by lobular carcinoma [34]. In the city of Lampang, cancer stage was reported as local, regional and distant in 29%, 50% and 10% of cases respectively [34]. In Bangkok, the distributions of cancer stage was known in 54% of cases and of these, 20% were reported as having local involvement while 28% and six percent had regional and distant involvement respectively [34].

Lertsanguansinchai *et al.* showed 93% (370/399) of cases were IDC with 41% (162/399) reported as poorly differentiated [35]. A retrospective review of 357 BC patients at Chulalongkorn University, Bangkok reported a mean age of 50 years with Stages one, two and IIIA reported in 11%, 80% and 10% of cases respectively [36].

##### 3.4.4.3. Hormone receptor status

Lertsanguansinchai *et al.* found 53% (213/399) of patients were ER+ while 42% (160/380) were PR+ [35]. Futhermore, 36% were ER+PR+, 16% were ER+PR−, 6% were ER−PR+, and 42% were ER−PR− [35]. Post menopausal women had a higher proportion of ER+ tumours while those that were reported as being larger, poorly differentiated, advanced stage or had greater lymph node positivity were more likely to be ER and PR negative [35].

#### 3.4.5. Philippines

##### 3.4.5.1. Background

The incidence of BC in Filipino women is amongst the highest in Asia [37]. Redaniel *et al.* reported that Philippine residents presented with BC at a mean age of 51 years [38].

##### 3.4.5.2. Tumour biology

De Leon Matsuda *et al.* reported on 294 cases and showed 49% (80/162) had nuclear grade two tumours while 41% (67/162) had nuclear grade three tumours (Table 2) [22]. Laudico *et al.* reported on the distribution of clinical cancer stage for the year 2002 and found 5% (8/159) of patients had stage one disease, 46% (73/159) had stage two disease, 14% (23/159) had stage three disease, and 8% (13/159) had stage four disease [39]. The distribution of cancer stage had not changed significantly between 1993 and 2002 [39].

##### 3.4.5.3. Hormone receptor status

De Leon Matsuda *et al.* found 59% of women (76/128) had ER+ tumours [22]. Uy *et al.* found the frequency of hormone receptor positive tumours increased from 59% to 69% following the implementation of tissue specimen fixation procedures [39].

#### 3.4.6. Indonesia

##### 3.4.6.1. Background

Aryandono *et al.* reported on 245 BC patients and found that 62% were between the ages of 40 to 59 with 19% under the age of 40 (Table 2) [23].

##### 3.4.6.2. Tumour biology

Ninety five percent of cases were IDC [23]. 52% (116/223) were reported as high grade, 44% (98/223) were intermediate grade and 4% (9/223) were low grade [23]. 60% (116/194) of tumours were greater than 20 mm in size while 21% (41/194) were larger than 50 mm [23]. Data on pathological cancer stage is reported in (Table 2) [23]. Distant metastases were reported in 18% (32/180) of cases. [23]. A high mitotic and MIB-1 (Ki67) index was reported in 66% (143/218) and 70% (130/186) of cases respectively [23]. Using multivariate analysis the authors found that the most significant prognostic factors for overall survival was lymph node status followed by clinical stage [23].

##### 3.4.6.3. Hormone receptor status

Aryandono *et al.* found 52% (124/238) of cases were ER+, 48% (110/227) were PR+, 64% (136/212) were positive for c-erbB2 (Her-2) and 55% (112/202) were p53 positive. [40] Forty percent (90/226) of tumours were ER+/PR+, 12% (27/226) were ER+/PR−, 8% (19/226) were ER−/PR+ and 40% (90/226) were ER−PR− [40].

#### 3.4.7. Vietnam

##### 3.4.7.1. Background

Lin *et al.* compared the average annual age adjusted incidence for *in situ* and invasive BC for Vietnamese women living in America for the time period 1988–1992 [41]. They found the rate for Vietnamese women was 35 per 100,000 [41].

##### 3.4.7.2. Tumour biology

Using American data, Vietnamese women (n = 280) presented at a mean age of 51 years with a high frequency of poorly differentiated tumours (31%) [41]. Regional involvement was similar amongst Vietnamese (26%) and non-Hispanic white women (24%) [41].

Williams *et al.* compared the biological differences of triple negative BC from 34 Vietnamese women and 56 women from the United States [42]. The mean age for both groups was 53 years with Vietnamese women having smaller tumours (32 mm) and fewer grade three cancers [62% (21/34)] [42]. Vietnamese women had a high frequency of ductal carcinoma at 91% (31/34) with no cases of medullar carcinoma reported (0/34)] [42].

##### 3.4.7.3. Hormone receptor status

In a sample of 236 Vietnamese women, Lin *et al.* found that approximately half of cases had ER+ (55%) and PR+ (48%) tumours [41]. Williams *et al.* found 73% (24/33) of tumours had expression of CK18, 61% (20/33) for EGFR; 82% (28/34) for CK8 and 88% (28/32) for P-cadherin [42].

#### 3.4.8. Malaysia

##### 3.4.8.1. Background

Hisham *et al.* found the median age at diagnosis was 44 years for Malay women compared to 50 and 53 years for Chinese and Indian women residing in Malaysia respectively [43]. 50% of cases were under the age of 50 with 17% below the age of 40 [43].

##### 3.4.8.2. Kuala Lumpur

###### Tumour biology

Malay women have lower incidence rates of BC compared to Chinese and Indian women, however they present with larger tumours with more advanced stage disease [44]. Ong *et al.* reported on 385 cases of BC without distant metastasis of which 61% of patients were Chinese [24]. The mean tumour diameter was 37 mm with grade two tumours reported in 53% (150/284) of cases, while 36% (103/284) were grade three and 11% (31/284) were grade one [24]. Lymphovascular involvement was noted in 33% (71/214) of cases [24]. When patients with stage four disease were also considered Malay patients were statistically more likely to present at a younger age, have larger tumours (>50 mm), higher disease stage (Stages three or four) with a greater frequency of lymph node involvement [24]. Hisham *et al.* reported on 774 new cases with the median age among the three ethnic groups of 50 years [43]. Furthermore, 50% to 60% of subjects had clinical stage three or four disease of which only 5% were detected through mammography screening [43]. A mean tumour size of 54 mm was reported (range 1–200 mm). The same authors had reported on a further 752 new cases and subsequently found a mean tumour size of 42 mm with 30–40% of cases with clinical stage three or four disease [43].

###### Hormone receptor status

Yip *et al.* reported that 56% of cases were ER+ [44]. Ong *et al.* reported that 60% (202/337) of cases were ER positive [24]. Al Joudi *et al.* reported on 383 cases of IDC and noted p53 expression in 30% of cases [45]. Furthermore, p53 expression was significantly correlated with patient age and clinical grade.

##### 3.4.8.3. Sabha

###### Tumour biology

Leong *et al.* found that IDC accounted for 84% (157/186) of tumours of which 59% (107/180) were grade two, 27% (49/180) were grade three and 13% (24/180) were grade one [25]. Details of cancer stage can be found in Table 2 [25].

###### Hormone receptor status

ER+ tumours were reported in 59% (110/186) of cases while 55% (102/186) were PR positive [25].

#### 3.4.9. Papua New Guinea

##### 3.4.9.1. Background

Halder *et al.* reported on 790 cases of BC and found that 84% of women were 54 years or younger with 56% under the age of 45 [46]. The age-standardized incidence was 6.9 per 100 000 [46].

##### 3.4.9.2. Tumour biology

Halder *et al.* found that IDC was present in 85% of specimens [46]. Of 163 tumours, 48% (78/163) measured between 60–100 mm, 45% (74/163) measured 30–50 mm and four percent (7/163) were greater than 100 mm [46]. Lymph node involvement was observed in 75% (185/247) of cases [46].

##### 3.4.9.3. Hormone receptor status

Pip *et al.* examined 26 BC specimens and found 81% of affected cases were premenopausal [47]. The same author found 54% (14/26) were ER−PR−, 38% (10/26) were ER-PR+, and 4% (1/26) were each ER+/PR− and ER+/PR+ [47].

## 4. Discussion

This review has shown that females with BC in the developing world have an early peak age at presentation with large and aggressive tumours. Distant metastases are frequently present at the time of diagnosis. Over half the women from the Asian subcontinent have lymph node metastases at first presentation with most cases having either grade two or three tumours. The rise in BC incidence amongst South East Asian countries exceeds that of the western world, with women from Malaysia and Philippines frequently presenting with stage three or four cancer. African women have a higher frequency of triple negative tumours. Over half of Middle Eastern women have lymph node involvement at the time of diagnosis. Despite experiencing a lower incidence compared to the Ashkenazi Jewish population, Palestinian women have poorer five-year survival outcomes. The majority of women from Mexico and South America have stage two or three disease whilst over sixty percent of women from Eastern Europe have either stage one or stage two diseases.

BC has long been considered a disease predominantly affecting affluent nations with the highest incidence rates reported in North America and Western Europe [48,49]. This is thought to be, at least partly, related to a greater prevalence of BC risk factors and the detection of early stage cancer through breast screening programmes [49,50]. Conversely, a lower incidence pattern is reported in developing nations such as those in Asia and Africa [48,49]. This is probably partially related to an under-reporting of cases due to the absence of cancer registries as well as a lower prevalence of risk factors for the development of BC [49,50]. Despite this the majority of BC deaths are reported in developing countries which have a higher mortality-to-incidence ratio [37,48]. For example the incidence to mortality ratio in South East Asia is 0.46 compared to 0.19 in North America [49].

In 2002, there were approximately 1.38 million new cases of BC worldwide and by 2020 this is expected to escalate to 1.7 million [48,51]. Furthermore, a projected 50% increase in BC mortality is anticipated worldwide, however this value is expected to be higher (58%) in developing nations [50]. Increasing life expectancy as well as changes in BC risk factor profile amongst developing nations are thought to contribute to the overall rise in BC incidence in the future.[50]. In urban Shanghai, the incidence of BC has increased by 50% [52]. In Mexico, BC is the leading cause of female cancer death whilst amongst the urban centres of India, it has surpassed cervical cancer as the leading female cancer [12,53]. The impact of ‘westernisation’ whereby a previously unexposed population are exposed to changes in risk profile (cohort effect) may partially be responsible for the increased BC risk observed [54]. Evidence to support this originates from migration studies which show a higher risk of BC in women who had migrated from Japan to Northern America [55]. Furthermore, data from the Shanghai BC study have identified similar hormonal and reproductive risk factors for the development of BC to that observed in the developed world which included earlier age at menarche, nulliparity later age at first live pregnancy and menopause and a lack of breastfeeding to be prevalent amongst affected cases [56,57]. Similar patterns have also been observed in Malaysia and other parts of the developing world [58,59].

The advanced nature of BC in the developing world has largely been attributed to the delays in seeking medical attention [60,61]. The reasons for this are multi factorial and include a lack of breast screening services combined with socioeconomic, cultural and political factors that underpin a propensity for women to present with advanced cancer [61,62]. Thongsuksai *et al.* had reported that 25% of BC patients from Thailand had waited 12 weeks from the recognition of symptoms before seeking medical advice, with similar patterns of behaviour observed amongst women from Iran (43%), Columbia (20%) and Peru (67%) [63-66]. Fears amongst patients and their families were identified as barriers to preventing timely access to early detection methods in Mexico [67]. Amongst Iranian women, a lack of knowledge regarding the necessity of such visits, fear, negligence, lack of access to physicians, and poverty were cited as the main reasons for delay [64]. The lack of BC education and the presence of other competing causes of morbidity and mortality means the community knowledge of BC is relatively limited [68]. Cultural factors are also influential, with affected cases only presenting to medical services once homeopathic and alternate therapies have been exhausted [25]. For example, Ajekigbe *et al.* identified preference for prayer houses or spiritual healing homes in 14% of cases with delayed presentation [69]. Errico *et al.* have shown that some women describe a sense of guilt of bringing “bad genes” into the family and are made to feel isolated by their communities over fears of ‘spreading’ their illness [70]. Women also carry fears over BC treatment. Ajekigbe *et al.* found that amongst Nigerian women, the most common reason for delayed presentation (45%) was fear of mastectomy with similar reasons identified by women from Pakistan [69,71].

The quality of treatment for BC in the developing world is highly variable. Agarawal *et al.* has described reoperation rates of up to 40% in certain sites in India following suboptimal surgery [68]. Furthermore, imperfections in pathological reporting of tumours have also been reported [72]. Inadequate tissue fixation has important consequences on the interpretation of hormone receptor status and hence treatment with hormonal type therapies [39]. In addition the ability to offer breast conserving surgery in developing countries is limited by the advanced nature of disease combined with a lack of radiotherapy services. A study from Delhi found only 11% of patients underwent breast conserving surgery due to the lack of radiotherapy services [73]. El Saghir *et al.* reported a total of 84 radiation therapy centres amongst all Arab countries combined compared to 1875 in the USA, despite the population numbers for the two areas being equivalent (approx 300 million) [74]. Finally, the financial costs also influence a women's decision to proceed with treatment as the costs of clinical visits and treatments have to be self funded.

The low peak age of incidence in the developing world may be at least partially explained by a lower overall life expectancy observed in developing countries [75]. Gukas *et al.* reported that only 5% of the Nigerian population were greater than 60 years of age compared to 21% of the British population [76]. Furthermore, the age standardised rate of BC was not higher amongst the Nigerian population. In fact, for both populations the age standardised rate was greater for women above the age of 50 [76]. In addition, it is well known that BC amongst premenopausal women tends to display poorer tumour characteristics [77,78]. Thus the earlier mean peak age at presentation may partially account for the aggressive pattern of cancer observed in much of the developing world.

Although increased parity has typically been associated with reduced risk of BC, Palmer *et al.* described the ‘dual effect of pregnancy’ and found African American women who had more than four children prior to the age of 45 years had a greater risk of BC and this was associated with a protective effect in women greater than 45 [79]. The role of increased parity (>3 live births) and increased BC risk amongst younger African women (but not white American women) was also noted by Hall *et al.*, although the results were deemed not significant [80]. Okobia *et al.* found increased parity (>4) carried an overall protective effect in a case control study of 250 Nigerian women with BC where the mean age of cases and controls was 46 and 47 respectively [81]. In contrast, Adebamowo *et al.* found a significant difference in the mean number of pregnancies in affected cases compared to controls also amongst Nigerian women [59].

A number of studies from Africa and the Middle East report a greater frequency of triple negative tumours. This has previously been related to poor tissue preservation [82]. Uy *et al.* reported a 10% increase in the number of hormone receptor positive tumours at the Philippines General Hospital after specific tissue fixation procedures were implemented [39]. Adawambabo *et al.* found no difference between ER/PR receptor status and age of Nigerian BC patients, although Awadelkarim *et al.* found a mean difference of 12 years between Sudanese and Italian cases positive for ER [60,82]. The Californian BC study showed premenopausal African American women had a greater frequency of basal like tumours (triple negative tumours) compared to non-African American women of any age [83]. Thus the lower frequency of hormone positive receptors reported amongst women living in Africa or other developing countries may reflect a combination of poor tissue fixation, patient age and possible differences in biology.

The aim of this study was to summarise the biological features of BC form developing countries. Due to the amount of detail already presented in this series we did not compare these findings to first world countries. For the same reason we did not elaborate on the management and treatment of BC in developing countries as discussion on this topic can be found elsewhere [50]. There were however some limitations to this report. A number of studies were based on the findings from single institutions within each country's largest city. Thus the BC characteristics of the country were inferred based on the results from a single study which reflect cases that, on average, have the highest standard of living. This is also a limitation of some National Cancer registries as the true BC incidence, mortality and biological characteristics from many of the smaller towns and villages remain largely unknown. As such we have incorporated the findings from World Health Organisation GLOBOCAN database which provides an estimate of BC incidence and mortality around the world including developing countries. One of the limitations that hindered valid comparisons between developing counties related to a lack of standardised reporting of data of BC characteristics. For example, some centres reported on tumour size and cancer stage based on clinical rather than pathological values while not all centres had reported on hormone receptor status. As a consequence some of the outcomes reported in this review were incomplete due to the variability in the data recorded. There are a limited number of studies which have assessed the contribution of genetic mutations to the development of BC within the context of the developing world. The lack of specialised laboratories, genetic reporting services and personnel amongst the developing world renders it difficult to elicit whether true genetic differences exist within ethnic groups. The role of socioeconomic factors is associated with poor prognosis and was not accounted for in this study. Finally, we were only able to review studies and abstracts written in English.

The global BC health initiative group have devised guidelines aimed at risk factor modification, early diagnosis, treatment and management of BC for developing countries [50]. With an increasing lifespan amongst individuals living in the developing world coupled with changes in risk factor profiles such as obesity and child bearing practises, BC incidence rates will continue to climb and a shifting trend from pre to post menopausal BC may occur [50,54]. Mammography remains the gold standard for early detection of BC [84]. However, the cost of establishing and maintaining such a programme may not be feasible in much of the developing world. Furthermore, mammography screening amongst pre menopausal women and the associated risks of high false positive tests would impose difficulties amongst developing countries. While alternative screening approaches such as self breast examinations have not been shown to reduce mortality in two randomised trials, the use of clinically trained professionals in performing clinical breast examinations in Egypt and India has shown encouraging results [85-89]. The importance of educating women of the symptoms, risk factors as well as addressing perceptions/misconceptions in a culturally sensitive manner remains essential [90].

## 5. Conclusions

BC in the developing world is characterised by an early peak age of onset with aggressive biological characteristics. There are a limited number of detailed studies in this field with significant variability of outcomes reported. Further research into the molecular and biological features as well as genetic determinants into BC development is certainly warranted. Importantly, public health initiatives that improve BC awareness address amenable risk factors and allow for the early detection of BC will be essential in addressing the outcome inequalities that currently exist.

## Figures and Tables

**Table 1.** Asian subcontinent.

**Country**	**N**	**Age (yrs) at presentation**	**Tumour size (mm)**	**Histology**		**Grade**			**LN+**	**ER+**	**PR+**
		**Mean/Median values**		**IDC**	**ILC**	**1**	**2**	**3**			
**Pakistan**											
Bhurgri *et al.* [2]	680	48	-	92%	1%	20%	59%	11%	56%	-	-
Azizun-Nisa *et al.* [3]	150	48	35% >50	85%	-	-	55%	-	71%	33%	25%
Usmani *et al.* [4]	1201	Peak 30–39	66% >50	-	-	-	-	58%	73%	-	-
Siddiqui *et al.* [5]	572	48	80% >20	81%			65%	24%	-	-	-
Sharif *et al.* [6]	535	48	44	90%	-	-	68%	-	65%	72%	63%
**India**											
Saxena *et al.* [7]	569	48	-	88%	4%	-	-	-	80%	-	-
Dinshaw *et al.* [8]	1022	43	30	92%	2%	2%	26%	70%	39%	33%	41%
**Sri Lanka**											
Lokuhetty *et al.* [9]	814	52	58% (20–50)	86%	8%	23%	52%	25%	41%	32%	-
Mudduwa *et al.* [10]	151	53	40	-	-	15%	36%	49%	58%	46%	48%

- Information not available.

**Table 2.** South East Asia.

**Country**	**N**	**Age**	**Histology**		**Stage**				**LN**
			**IDC**	**ILC**	**1**	**2**	**3**	**4**	**+**
China									
Liu *et al.* [18]	1678	46-47	-	-	16%	-	17%	-	46%
Gao *et al.* [19]	144	52	-	-	58% *		42% *		40%
Taiwan									
Lin *et al.* [20]	1028	<50	92%	3%	34%	44%	17%	5%	45%
South Korea									
Ahn *et al.* [21]	9667	47	86%	2%	36%		55%		-
Philippines									
De Leon Matsuda *et al.* [22]	294	44	87%	1%	3%	32%	52%	10%	67%
Indonesia									
Aryandono *et al.* [23]	223	-	95%	-	15%	49%	37%	-	62%
Malaysia									
Ong *et al.* [24]	385	50	-	-	24%	56%	20%	#	51%
Leong *et al.* [25]	186	51	84%	3%	13%	30%	37%	16%	-

* Clinical stage;

# Patients with stage four cancer were excluded;

- Information not available.

## References

[1] Bhikoo R., Srinivasa S., Yu T.C., Moss D., Hill A.G. (2011). Systematic review of breast cancer biology (Part 1): Africa, the Middle East, Eastern Europe, Mexico, the Caribbean and South America. Cancers.

[2] Bhurgri Y., Kayani N., Faridi N., Pervez S., Usman A., Bhurgri H., Malik J., Bashir I., Bhurgri A., Hasan S.H., Zaidi S.H. (2007). Patho-epidemiology of breast cancer in Karachi ‘1995-1997’. Asian Pac. J. Cancer Prev..

[3] Bhurgri Y., Raza F., Kayani N., Azizsun-Nisa (2008). Comparison of ER, PR and HER-2/neu (C-erb B 2) reactivity pattern with histologic grade, tumor size and lymph node status in breast cancer. Asian Pac. J. Cancer Prev..

[4] Usmani K., Khanum A., Afzal H., Ahmad N. (1996). Breast carcinoma in Pakistani women. J. Environ. Pathol. Toxicol. Oncol..

[5] Siddiqui M.S., Kayani N., Sulaiman S., Hussainy A.S., Shah S.H., Muzaffar S. (2000). Breast carcinoma in Pakistani females: A morphological study of 572 breast specimens. J. Pak. Med. Assoc..

[6] Sharif M.A., Mamoon N., Mushtaq S., Khadim M.T. (2009). Morphological profile and association of HER-2/neu with prognostic markers in breast carcinoma in Northern Pakistan. J. Coll. Phys. Surg. Pak..

[7] Saxena S., Rekhi B., Bansal A., Bagga A., Chintamani, Murthy N.S. (2005). Clinico-morphological patterns of breast cancer including family history in a New Delhi hospital, India-a cross-sectional study. World J. Surg. Oncol..

[8] Dinshaw K.A., Budrukkar A.N., Chinoy R.F., Sarin R., Badwe R., Hawaldar R., Shrivastava S.K. (2005). Profile of prognostic factors in 1022 Indian women with early-stage breast cancer treated with breast-conserving therapy. Int. J. Radiat. Oncol. Biol. Phys..

[9] Lokuhetty M.D., Ranaweera G.G., Wijeratne M.D., Wickramasinghe K.H., Sheriffdeen A.H. (2009). Profile of breast cancer in a group of women in a developing country in South Asia: Is there a difference?. World J. Surg..

[10] Mudduwa L. (2009). Quick score of hormone receptor status of breast carcinoma: Correlation with the other clinicopathological prognostic parameters. Indian J. Pathol. Microbiol..

[11] Aziz S.A., Pervez S., Khan S., Kayani N., Rahbar M.H. (2001). Relationship of p53 expression with clinicopathological variables and disease outcome: A prospective study on 315 consecutive breast carcinoma patients. Malays J. Pathol..

[12] Indian Council of Medical Research. National Cancer Registry Programme (2001–2004). Consolidated Report of Population Based Cancer Registries. http://www.ncrpindia.org/.

[13] Desai S.B., Moonim M.T., Gill A.K., Punia R.S., Naresh K.N., Chinoy R.F. (2000). Hormone receptor status of breast cancer in India: A study of 798 tumours. Breast.

[14] Statistics Home Page. http://www.ncisl.lk/statistic.php/.

[15] Ratnatunga N., Liyanapathirana L.V. (2007). Hormone receptor expression and Her/2neu amplification in breast carcinoma in a cohort of Sri Lankans. Ceylon Med. J..

[16] Pradhan M., Dhakal H.P. (2008). Study of breast lump of 2246 cases by fine needle aspiration. JNMA J. Nepal. Med. Assoc..

[17] Sharma A., Bandari R., Gilbert D., Sharma A.K. (2005). Benign and malignant breast disease presenting to Bhaktapur Cancer Hospital. Kathmandu Univ. Med. J. (KUMJ).

[18] Liu H., Xun P., Chen K.X., Li H.X., Hao X.S. (2007). The trend of clinical characteristics and prognosis of women's breast cancer 1981-2000. Zhonghua Yi Xue Za Zhi.

[19] Gao R.J., Bao H.Z., Yang Q., Cong Q., Song J.N., Wang L. (2005). The presence of serum anti-p53 antibodies from patients with invasive ductal carcinoma of breast: Correlation to other clinical and biological parameters. Breast Cancer Res. Treat..

[20] Lin C.H., Liau J.Y., Lu Y.S., Huang C.S., Lee W.C., Kuo K.T., Shen Y.C., Kuo S.H., Lan C., Liu J.M. (2009). Molecular subtypes of breast cancer emerging in young women in Taiwan: Evidence for more than just westernization as a reason for the disease in Asia. Cancer Epidemiol. Biomarker. Prev..

[21] Ahn S.H., Yoo K.Y. (2006). Korean Breast Cancer Society. Chronological changes of clinical characteristics in 31,115 new breast cancer patients among Koreans during 1996-2004. Breast Cancer Res. Treat..

[22] De Leon Matsuda M.L., Liede A., Kwan E., Mapua C.A, Cutiongco C.M., Tan A., Borg A., Narod S.A. (2002). BRCA1 and BRCA2 mutations among breast cancer patients from the Philippines. Int. J. Cancer.

[23] Aryandono T., Harijadi S. (2006). Survival from operable breast cancer: Prognostic factors in Yogyakarta, Indonesia. Asian Pac. J. Cancer Prev..

[24] Ong T.A., Yip C.H. (2003). Short-term survival in breast cancer: The experience of the University of Malaya Medical Centre. Asian J. Surg..

[25] Leong B.D., Chuah J.A., Kumar V.M., Yip C.H. (2007). Breast cancer in Sabah, Malaysia: A two year prospective study. Asian Pac. J. Cancer Prev..

[26] Zheng Y. Cancer Surveillance in Shanghai, China.

[27] Zhang T., Tu X., Xu W. (1998). A study of prognostic factors in breast cancer: Histological grading. Zhonghua Yi Xue Za Zhi.

[28] Yuan Z.Y., Wang S.S., Gao Y., Su Z.Y., Luo W.B., Guan Z.Z. (2008). Clinical characteristics and prognosis of triple-negative breast cancer: A report of 305 cases. Ai Zheng.

[29] Taiwan Cancer Registry Cancer Incidence and Mortality Rates in Taiwan 2006 Home Page. http://tcr.cph.ntu.edu.tw/main.php?Page=N2/.

[30] National Cancer Centre Cancer Facts and Figures in the Republic of Korea 2009 Home Page. http://www.ncc.re.kr/english/cyber/publi01.jsp/.

[31] Choi D.H., Shin D.B., Lee M.H., Dhandapani D., Carter D., King B.L., Haffty B.G. (2003). A comparison of five immunohistochemical biomarkers and HER-2/neu gene amplification by fluorescence in situ hybridization in white and Korean patients with early-onset breast carcinoma. Cancer.

[32] Son B.H., Kwak B.S., Kim J.K., Kim H.J., Hong S.J., Lee J.S., Hwang U.K., Yoon H.S., Ahn S.H. (2006). Changing patterns in the clinical characteristics of Korean patients with breast cancer during the last 15 years. Arch. Surg..

[33] Rhee J., Han S.W., Oh D.Y, Kim J.H., Im S.A., Han W., Park I.A., Noh D.Y., Bang Y.J., Kim T.Y. (2008). The clinicopathologic characteristics and prognostic significance of triple-negativity in node-negative breast cancer. BMC Cancer.

[34] Khuhaprema T., Srivatanakul P., Sriplung H., Wiangnon S., Sumitsawan Y., Attasara P. (2007). Cancer in Thailand Vol. IV, 1998-2000.

[35] Lertsanguansinchai P., Chottetanaprasith T., Chatamra K., Sampatunukul P., Wannakrairot P., Rojpornpradit P., Shotelersuk K., Lertbutsayanukul C., Boonjunwetwat D., Vajragupta L. (2002). Estrogen and progesterone receptors status in Thai female breast cancer patients: An analysis of 399 cases at King Chulalongkorn Memorial Hospital. J. Med. Assoc. Thai..

[36] Lertsanguansinchai P., Lertbutsayanukul C., Chatamra K., Shotelersuk K., Voravud N, Khorprasert C. (2004). Pattern of local-regional recurrence in patient with early breast cancer after mastectomy: An analysis of 357 cases at King Chulalongkorn Memorial Hospital. J. Med. Assoc. Thai..

[37] Curado M.P., Edwards B., Shin H.R. (2007). Cancer Incidence in Five Continents, Vol. IX.

[38] Redaniel M.T., Laudico A., Mirasol-Lumague M.R., Gondos A., Pulte D., Mapua C., Brenner H. (2009). Cancer survival discrepancies in developed and developing countries: Comparison between the Philippines and the United States. Br. J. Cancer.

[39] Laudico A, Redaniel M.T., Mirasol-Lumague M.R., Mapua C.A., Uy G.B., Pukkala E., Pisani P. (2009). Epidemiology and clinicopathology of breast cancer in metro Manila and Rizal Province, Philippines. Asian Pac. J. Cancer Prev..

[40] Aryandono T., Harijadi S. (2006). Hormone receptor status of operable breast cancers in Indonesia: Correlation with other prognostic factors and survival. Asian Pac. J. Cancer Prev..

[41] Lin S.S., Phan J.C., Lin A.Y. (2002). Breast cancer characteristics of Vietnamese women in the Greater San Francisco Bay Area. West J. Med..

[42] Williams D.J., Cohen C., To T.V., Page A.J., Lawson D., Sussman Z.M., Nassar A. (2009). Triple-negative breast carcinoma in women from Vietnam and the United States: Characterization of differential marker expression by tissue microarray. Hum. Pathol..

[43] Hisham A.N., Yip C.H. (2004). Overview of breast cancer in Malaysian women: A problem with late diagnosis. Asian J. Surg..

[44] Yip C.H., Taib N.A., Mohamed I. (2006). Epidemiology of breast cancer in Malaysia. Asian Pac. J. Cancer Prev..

[45] Al-Joudi F.S., Iskandar Z.A., Rusli J. (2008). The expression of p53 in invasive ductal carcinoma of the breast: A study in the North-East States of Malaysia. Med. J. Malaysia.

[46] Halder A., Morewya J., Watters D.A. (2001). Rising incidence of breast cancer in Papua New Guinea. ANZ J. Surg..

[47] Pip A., Watters D., Murthy D., Wood N., Donnelly P. (1998). Hormone-receptor status of breast cancer in Papua New Guinea. Lancet.

[48] Ferlay J.S.H., Bray F., Forman D., Mathers C., Parkin D.M. GLOBOCAN 2008, Cancer Incidence and Mortality Worldwide: IARC CancerBase No 10 [Internet]. http://globocaniarcfr/.

[49] Parkin D.M., Fernández L.M. (2006). Use of statistics to assess the global burden of breast cancer. Breast J..

[50] Anderson B.O., Yip C.H., Smith R.A., Shyyan R., Sener S.F., Eniu A., Calson R.W., Azavedo E., Harford J. (2008). Guideline implementation for breast healthcare in low-income and middle-income countries: Overview of the Breast Health Global Initiative Global Summit 2007. Cancer.

[51] The Lancet (2009). Breast cancer in developing countries. Lancet.

[52] Jin F., Devesa S.S., Chow W.H., Zheng W., Ji B.T., Fraumeni J.F., Gao Y.T. (1999). Cancer incidence trends in urban shanghai, 1972-1994: An update. Int. J. Cancer.

[53] Knaul F.M., Nigenda G., Lozano R., Arreola-Ornelas H, Langer A., Frenk J. (2008). Breast cancer in Mexico: A pressing priority. Reprod. Health Matters.

[54] Porter P. (2008). “Westernizing” women's risks? Breast cancer in lower-income countries. N. Engl. J. Med..

[55] Ziegler R.G., Hoover R.N., Pike M.C., Hildesheim A., Nomura A.M., West D.W., Wu-Williams A.H., Kolonel L.N., Horn-Ross P.L., Rosenthal J.F. (1993). Migration patterns and breast cancer risk in Asian-American women. J. Natl. Cancer Inst..

[56] Gao Y.T., Shu X.O., Dai Q., Potter J.D., Brinton L.A., Wen W., Sellers T.A., Kushi L.H., Ruan Z., Bostick R.M. (2000). Association of menstrual and reproductive factors with breast cancer risk: Results from the Shanghai Breast Cancer Study. Int. J. Cancer.

[57] McPherson K., Steel C.M., Dixon J.M. (2000). ABC of breast diseases. Breast cancer-epidemiology, risk factors, and genetics. BMJ.

[58] Norsa'adah B., Rusli B.N., Imran A.K., Naina I., Winn T. (2005). Risk factors of breast cancer in women in Kelantan, Malaysia. Singapore Med. J..

[59] Adebamowo C.A., Adekunele O.O. (1999). Case-controlled study of the epidemiological risk factors for breast cancer in Nigeria. Br. J. Cancer.

[60] Awadelkarim K.D., Arizzi C., Elamin E.O., Hamad H.M., De Blasio P., Mekki S.O., Osman I., Buinno I., Elwali N.E., Mariani-Costantini R. (2008). Pathological, clinical and prognostic characteristics of breast cancer in Central Sudan *versus* Northern Italy: Implications for breast cancer in Africa. Histopathology.

[61] Hisham A.N, Yip C.H. (2003). Spectrum of breast cancer in Malaysian women: Overview. World J Surg.

[62] Adesunkanmi A.R., Lawal O.O., Adelusola K.A., Durosimi M.A. (2006). The severity, outcome and challenges of breast cancer in Nigeria. Breast.

[63] Thongsuksai P., Chongsuvivatwong V., Sriplung H. (2000). Delay in breast cancer care: A study in Thaiwomen. Med. Care.

[64] Harirchi I., Ghaemmaghami F., Karbakhsh M., Moghimi R., Mazaherie H. (2005). Patient delay inwomen presenting with advanced breast cancer: An Iranian study. Public Health.

[65] Piñeros M., Sánchez R., Cendales R., Perry F., Ocampo R. (2009). Patient delay among Colombian women with breast cancer. Salud Publica Mex.

[66] Contreras Zaravia N.R., Valdeiglesias Cabrera N., Saco Méndex S., Majía Palomino O. (2000). Demora en el diagnóstico de cáncer de mama: Factores de la paciente. Hospital EsSalud Cusco 1986-1999. SITUA.

[67] Nigenda G., Caballero M., González-Robledo L.M. (2009). Access barriers in early diagnosis of breastcancer in the Federal District and Oaxaca. Salud Publica Mex.

[68] Agarwal G., Ramakant P., Forgach E.R., Rendón J.C., Chaparro J.M., Basurto C.S., Margaritoni M. (2009). Breast cancer care in developing countries. World J. Surg..

[69] Ajekigbe A.T. (1991). Fear of mastectomy: The most common factor responsible for late presentation of carcinoma of the breast in Nigeria. Clin. Oncol..

[70] Errico K.M., Rowden D. (2006). Experiences of breast cancer survivor-advocates and advocates in countries with limited resources: A shared journey in breast cancer advocacy. Breast J..

[71] Khan M.M., Jan M.A., Shah S., Begum H., Khan M.S. (2008). Breast Disease: Causes for delays in presentation. J. Med. Sci..

[72] Kuraparthy S., Reddy K.M., Yadagiri L.A, Yutla M., Venkata P.B., Kadainti S.V., Reddy R.P. (2007). Epidemiology and patterns of care for invasive breast carcinoma at a community hospital in Southern India. World J. Surg. Oncol..

[73] Raina V., Bhutani M., Bedi R., Sharma A., Deo S.V., Shukla N.K., Mohanti B.K., Rath G.K. (2005). Clinical features and prognostic factors of early breast cancer at a major cancer center in North India. Indian J. Cancer.

[74] El Saghir N.S., Shamseddine A.I., Geara F., Bikhazi K., Rahal B., Salem Z.M., Taher A., Tawil A., El Khatib Z., Abbas J. (2002). Age distribution of breast cancer in Lebanon: Increased percentages and age adjusted incidence rates of younger-aged groups at presentation. J. Med. Liban.

[75] United Nations United Nations Statistics Division Indicators on Health Home Page. http://unstats.un.org/unsd/demographic/products/socind/health.htm.

[76] Gukas I.D., Jennings B.A., Mandong B.M., Manasseh A.N., Harvey I., Leinster S.J. (2006). A comparison of the pattern of occurrence of breast cancer in Nigerian and British women. Breast.

[77] Klauber-DeMore N. (2005-2006). Tumor biology of breast cancer in young women. Breast Dis..

[78] Anders C.K., Hsu D.S., Broadwater G., Acharya C.R., Foekens J.A., Zhand Y., Wang Y., Marcom P.K., Marks J.R., Febbo P.G. (2008). Young age at diagnosis correlates with worse prognosis and defines a subset of breast cancers with shared patterns of gene expression. J. Clin. Oncol..

[79] Palmer J.R., Wise L.A., Horton N.J., Adams-Campbell L.L., Rosenberg L. (2003). Dual effect of parity on breast cancer risk in African-American women. J. Natl. Cancer Inst..

[80] Hall I.J., Moorman P.G., Millikan R.C., Newman B. (2005). Comparative analysis of breast cancer risk factors among African-American women and White women. Am. J. Epidemiol..

[81] Okobia M., Bunker C., Zmuda J., Kammerer C., Vogel V., Uche E., Anyanwu S., Ezeome E., Ferrell R., Kuller L. (2006). Case-control study of risk factors for breast cancer in Nigerian women. Int. J. Cancer.

[82] Adebamowo C.A., Famooto A., Ogundiran T.O., Aniagwu T., Nkwodimmah C., Akang E.E. (2008). Immunohistochemical and molecular subtypes of breast cancer in Nigeria. Breast Cancer Res. Treat..

[83] Carey L.A., Perou C.M., Livasy C.A., Dressler L.G., Cowan D., Conway K., Karaca G., Troester M.A., Tse C.K., Edmiston S. (2006). Race, breast cancer subtypes, and survival in the Carolina Breast Cancer Study. JAMA.

[84] Vainio H., Bianchini F. (2002). IARC Handbooks of Cancer Prevention. Vol. 7: Breast Cancer Screening.

[85] Weiss N. (2003). Breast cancer mortality in relation to clinical breast examination and breast self-examination. Breast J..

[86] Boulos S.G.M., Neguib S., Essam E., Youssef A., Costa A., Mittra A., Miller A.B. (2005). Breast screening in the emerging world: High prevalence of breast cancer in Cairo. Breast.

[87] Thomas D.B., Gao D.L., Ray R.M. (2002). Randomized trial of breast self-examination in Shanghai: Final results. J. Natl. Cancer Inst..

[88] Dinshaw K., Mishra G., Shastri S., Badwe R., Kerkar R., Ramani S., Thakur M., Uplap P., Kakade A., Gupta S. (2007). Determinants of compliance in a cluster randomised controlled trial on screening of breast and cervix cancer in Mumbai, India. 1. Compliance to screening. Oncology.

[89] Dinshaw K., Mishra G., Shastri S., Badwe R., Kerkar R., Ramani S., Thakur M., Uplap P., Kakade A., Gupta S. (2007). Determinants of compliance in a cluster randomised controlled trial on screening of breast and cervix cancer in Mumbai, India. 2. Compliance to referral and treatment. Oncology.

[90] Yip C.H., Smith R.A., Anderson B.O., Miller A.B., Thomas D.B., Ang E.S., Caffarella R.S., Corbex M., Kreps G.L, McTiernan A. (2008). Guideline implementation for breast healthcare in low- and middle-income countries: Early detection resource allocation. Cancer.

